# Supplementary material for: Isolation and Functional Characterization of Two CONSTANS-like 16 (MiCOL16) Genes from Mango
Source: Int J Mol Sci. 2022 Mar 12;23(6):3075. doi: 10.3390/ijms23063075 (PMC8951110; doi:10.3390/ijms23063075)
Supplement: Supplementary file 1 [file ijms-23-03075-s001.zip › ijms-1615170-supplementary.pdf]

Supplementary Material

Supplementary Table

Table S1. The following genes and their accession numbers were used.

Plant species	Gene names	Accession numbers
	AtCO	NM_121589
	AtCOL1	NM_121590
	AtCOL2	NM_111105
	AtCOL3	NM_201801
	AtCOL4	NM_122402
	AtCOL5	NM_125149
	AtCOL6	AY081541
	AtCOL7	NM_106047
	AtCOL8	NM_103803
Arabidopsis thaliana (At)	AtCOL9	NM_111644
	AtCOL10	NM_124200
	AtCOL11	NM_117613
	AtCOL12	NM_113084
	AtCOL13	NM_130356
	AtCOL14	NM_128910
	AtCOL15	NM_102570
	AtCOL16	NM_102355
Mangifera indica (Mi)	MiCOL16A	MW326761
	MiCOL16B	MW326762
